# Natural Mineral Waters and Metabolic Syndrome: Insights From Obese Male and Female C57BL/6 Mice on Caloric Restriction

**DOI:** 10.3389/fnut.2022.886078

**Published:** 2022-05-24

**Authors:** Laura Narciso, Andrea Martinelli, Flavio Torriani, Paolo Frassanito, Roberta Bernardini, Flavia Chiarotti, Cinzia Marianelli

**Affiliations:** ^1^Department of Food Safety, Nutrition and Veterinary Public Health, Istituto Superiore di Sanità, Rome, Italy; ^2^Centre for Animal Experimentation and Well-Being, Istituto Superiore di Sanità, Rome, Italy; ^3^Interdepartmental Center for Comparative Medicine, Alternative Techniques and Aquaculture, University of Rome “Tor Vergata,” Rome, Italy; ^4^Centre for Behavioural Sciences and Mental Health, Istituto Superiore di Sanità, Rome, Italy

**Keywords:** metabolic syndrome, obesity, C57BL/6 mice, animal model, natural mineral waters, gender differences

## Abstract

Metabolic syndrome (MetS) represents one of the greatest challenges to public health given its serious consequences on cardiovascular diseases and type 2 diabetes. A carbohydrate-restricted, low-fat diet is the current therapy for MetS. Natural mineral waters (NMWs) are known to exert beneficial effects on human health. Our primary objective was to shed light on the potential therapeutic properties of NMWs in MetS. A total of 125 C57BL/6 male and female mice were included in the study. Of these, 10 were left untreated. They were fed a standard diet with tap water throughout the study period, and stayed healthy. The remaining 115 mice were initially fed a high-calorie diet (HCD) consisting of a high-fat feed (60% of energy from fat) with 10% fructose in tap water, served *ad libitum* over a period of 4 months to induce MetS (the MetS induction phase). Mice were then randomly divided into six treatment groups and a control group, all of which received a low-calorie diet (LCD), but with a different kind of drinking water, for 2 months (the treatment phase). Five groups were each treated with a different kind of NMW, one group by alternating the five NMWs, and one group – the control group – was given tap water. Body weight and blood biochemistry were monitored over the 6-month trial. After 4 months, male and female mice on HCD developed obesity, hypercholesterolaemia and hyperglycaemia, although gains in body weight, total cholesterol, and blood glucose in males were greater than those observed in females (*P* < 0.0001). When combined with an LCD, the NMWs rich in sulphate, magnesium and bicarbonate, and the minimally mineralised one were the most effective in reducing the blood levels of total cholesterol, high-density lipoprotein (HDL) cholesterol, and glucose. Sex differences emerged during both the MetS induction phase and the treatment phase. These results suggest that NMWs rich in specific macronutrients, such as bicarbonate, sulphate and magnesium, and minimally mineralised water, in combination with an LCD, may contribute to controlling blood lipid and glucose levels in subjects with MetS. Further studies are needed to confirm these results and to extend them to humans.

## Introduction

Metabolic syndrome (MetS) is a cluster of multiple, interrelated metabolic features such as obesity, hyperglycaemia, hypertension, and dyslipidaemia (1). Worldwide, the prevalence of MetS ranges from 10 to 84% depending on age, gender, race, ethnicity, and definition of MetS (2). Due to its rising prevalence, and its association with an increased risk of cardiovascular disease, type 2 diabetes, non-alcoholic fatty liver disease, and cancer (liver, pancreas, breast, bladder, and prostate) (2), MetS is becoming a major public health concern. Insulin resistance, altered redox state, low grade pro-inflammatory state, hypercoagulable/prothrombotic state, and endothelial dysfunction are also characteristics of MetS (2, 3).

Although the aetiology of MetS is still not entirely clear, it is known to involve complex interactions between genetic, metabolic, and environmental factors (2). One of the primary causes of the current epidemic of obesity and related metabolic disorders is associated with the Western-style diet, which includes excessive intake of high-fat and high-sucrose foods. Lifestyle modifications that include limiting energy intake from total fats and sugars and engaging in regular physical activity, are key factors in reducing obesity and preventing related diseases (4). Because these traditional approaches strongly rely on the individual’s behaviour and motivation, they are often unsuccessful in clinical practice. Novel strategies for the prevention and treatment of metabolic diseases, and of obesity in particular, should be considered.

Water is the basic element of life. According to current European Community directives, natural mineral waters (NMWs) are defined as waters of underground origin that are protected from contamination and are microbiologically wholesome. In addition, each kind of NMW presents a specific, constant chemical composition, organoleptic characteristics and temperature, and possess health promoting properties (5–8). As demonstrated by studies on both animal models and humans, natural, mineral drinking waters possess unique therapeutic and preventive properties for a wide range of diseases affecting the respiratory tract, skin, liver, intestine, female reproductive system, and osteoarticular system (9). It has been reported that minerals/elements dissolved in water are more readily and easily absorbed (bioavailable) than those from food (10–12).

The beneficial health effects of NMWs have been documented mainly in the context of intestinal diseases. Bicarbonate waters are indicated for dyspepsia, irritable bowel syndrome, and functional disorders of the biliary tract because they are able to neutralise acid secretion and stimulate the secretions and motility of the digestive tract (13). Sulphate waters stimulate intestinal motility and are mainly indicated in cases of functional constipation (14). Sulphate-bicarbonate waters are used for biliary dyskinesia, biliary sand, and post-cholecystectomy syndrome (7, 15). Salt waters stimulate intestinal peristalsis and intestinal secretion of water and electrolytes (16). Sulphurous and bicarbonate waters are indicated in cases of diabetes in order to control glycaemia, polydipsia, and polyuria, reduce insulin requirements and neutralise metabolic acidosis in diabetic decompensation (7). In short, clinical studies have shown that mineral water drinking therapy can affect glucose and lipid metabolism, depending on the particular chemical/physical composition of the mineral water administered.

Twelve studies have thus far been conducted to investigate the effects of NMW consumption on MetS features in humans (17). Unfortunately, differences in the design of these studies – their duration, the diet administered, the population size and characteristics of enrolled subjects, the amount and composition of NMW consumed – make it difficult to draw definitive conclusions. Rodent models of MetS – a valuable tool for studying the major characteristics of this syndrome, such as obesity, hyperglycaemia, dyslipidaemia, diabetes, and low-grade inflammation (18, 19) – have been used to evaluate the protective effects of NMW ingestion against the induction of MetS in fructose-fed animals (20–22). To the best of our knowledge, however, no studies are available in the literature on the metabolic impact of NMWs in rodents with MetS.

The main objective of the study was to assess the effectiveness of the oral administration of NMWs, coupled with a low-fat, carbohydrate-restricted diet, on alleviating the signs of MetS in a diet-induced C57BL/6 mouse model of the syndrome. Specifically, we investigated the effect of mineral drinking waters in combination with a low-energy diet on body weight, and on glucose and lipid metabolism. Trends in body weight and blood biochemistry during both the MetS induction and NMW treatment phases are described.

We also looked into the appropriateness of including both male and female mice as models of MetS.

## Materials and Methods

### Mice

A total of 125 C57BL/6 mice of both sexes (*n* = 60 females and *n* = 65 males) purchased from Charles River Laboratories Italia Srl at 7–8 weeks of age, were included in the study. Animals were randomly housed in single-sex groups of two/three individuals per cage in a temperature-controlled room with a 12-h light/dark cycle in the animal facility of the Istituto Superiore di Sanità (Rome, Italy). All animals were provided with nesting material and cage enrichment.

After 1 week of adaptation to a standard diet (StD, 18.50% protein, 53.50% carbohydrate, and 3% fat) (4RF21, Mucedola Srl, Italy), ten mice (*n* = 5 females and *n* = 5 males, healthy untreated group) were kept on StD and provided with tap water *ad libitum*, over the entire 6-months. The remaining 115 mice entered the 6-month trial.

All procedures involving animals were reviewed and approved by the Italian Ministry of Health (Permit number 938/2018-PR-14/12/2018), and were complied with the ARRIVE guidelines. Animal care and treatment were conducted in accordance with the institutional guidelines and international laws and policies (Directive 2010/63/EU on the protection of animals used for scientific purposes).

### Experimental Design

**FIGURE 1:**
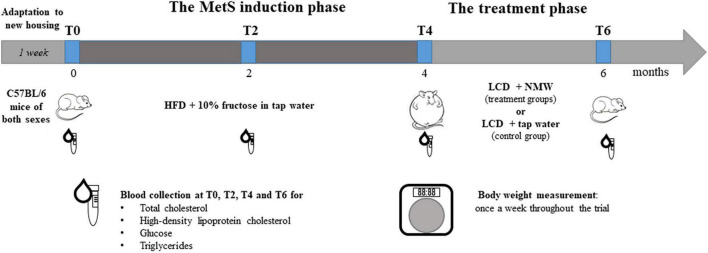
Experimental design. Both male and female C57BL/6 mice were initially fed a high-calorie diet (HCD) with 10% fructose in tap water over a period of 4 months to induce MetS (the MetS induction phase). Mice were then randomly divided into treatment groups and a control group, all of which received a low-calorie diet (LCD), but with a different kind of drinking water, for 2 months (the treatment phase). The treatment groups were each treated with a different kind of NMW, and one group – the control group – was given tap water. Body weight and blood biochemistry were monitored over the 6-month trial: before (T0) and after 1 (T1), 2 (T2), 3 (T3), and 4 (T4) months feeding on HCD, and after 2 months (T6) on LCD.

The 6-month trial is illustrated in Figure 1. In brief, after allowing 1 week for adaptation, the 115 mice were started on a high-calorie diet (HCD) for 4 months in order to induce MetS. Subsequently, the treatment phase was initiated, during which the 115 mice were put on a calorie-restricted diet, and treated with either NMW (mineral water-treated groups) or tap water (control group). Body weight and blood biochemistry were measured throughout the trial and analysed (see section “Body Weight and Blood Biochemistry Testing” below).

### Drinking Waters

Five kinds of natural, mineral drinking water commercially available in Italy, and the tap water distributed in Rome, Italy, were employed in the study.

According to the 2009/54/EC Directive and on the basis of their main mineral content, the five NMWs were classified as: sulphate-, calcium-, and magnesium-rich mineral water (SCMmw), bicarbonate-, sulphate-, and magnesium-rich mineral water (BSMmw), bicarbonate- and calcium-rich mineral water (BCmw), bicarbonate-, calcium-, potassium-, and magnesium-rich mineral water (BCPMmw), and light mineral water (lmw, with no main mineral). The above NMWs were administered during the treatment phase to the mineral water-treated groups.

The tap water used in this study is the water distributed by the ACEA Group to the residents of Rome. This drinking water originates from springs (97%) and wells (3%). It is chlorinated and monitored daily. Monitoring reports are available on the ACEA website.^1^ Tap water was administered to the healthy untreated group (10 mice), to all 115 trial animals during the MetS induction phase, and to only one of the treatment groups – the control group – during the treatment phase.

The physical and chemical characteristics of the kinds of drinking water used in the study are shown in Table 1: NMWs (as tested by an independent, government-appointed laboratory, and indicated on the water bottle labels) and tap water (as per the ACEA report).

**TABLE 1:** Characteristics of the kinds of drinking water used in the study.

Characteristics	SCMmw	BSMmw	BCmw	BCPMmw	lmw	Tap water
Bicarbonate (mg/L)	268	1,908	1,010	2,400	11	398
Sulphate (mg/L)	1,590	445	61	–	–	16.7
Magnesium (mg/L)	101	394	16.5	61.3	–	18.9
Calcium (mg/L)	580	48	323	212.2	3.3	104
Potassium (mg/L)	–	16.1	3.85	83.4	–	0.98
Sodium (mg/L)	14	160	19.6	–	1.5	4.1
Chlorides (mg/L)	–	46	18.5	–	–	6.4
Nitrates (mg/L)	<0.5	0.6	0.9	–	0.88	2.99
Fluoride (mg/L)	–	0.19	0.3	–	<0.10	0.12
pH	7.2	6.3	6.2	6.6	6.8	7.5
Electrical conductivity at 20°C (μS/cm)	2,300	2,760	1,325	4,060	25.5	571
Fixed residue at 180°C (mg/L)	2,470	2,128	987	3,002.3	22	408

*SCMmw, sulphate-, calcium-, and magnesium-rich mineral water; BSMmw, bicarbonate-, sulphate-, and magnesium-rich mineral water; BCmw, bicarbonate- and calcium-rich mineral water; BCPMmw, bicarbonate-, calcium-, potassium-, and magnesium-rich mineral water; lmw, light mineral water; tap water, drinking water distributed by the ACEA Group in Rome.*

### Trial

#### The Metabolic Syndrome Induction Phase

A total of 115 mice (*n* = 55 females and *n* = 60 males) were fed an HCD consisting of a high-fat feed (HFD, 23% protein, such as casein, 32.50% carbohydrate, such as sucrose and maltodextrin, and 34% fat, such as lard, and palm and soybean oils; PF4215, Mucedola Srl, Italy), which provided 60% of the energy from fat. This was served with 10% fructose (Fruttil) in tap water *ad libitum* over a period of 4 months. Body weight and blood biochemistry measurements were conducted to ascertain the development of MetS.

#### The Treatment Phase

Mice with diet-induced MetS of both sexes were randomly divided into seven groups (six treated with mineral water and one control group treated with tap water). We had intended to cluster *n* = 8 females and *n* = 8 males per group. However, due to a modest mortality rate (5/115, four cases of aggression in male mice and one case of ulcerative dermatitis in a female), group composition varied (see below).

Over the 2 months following the induction phase, all animals were fed a low-calorie diet (LCD, 16% protein, such as casein, 55.50% carbohydrate, such as sucrose and starch, and 2.5% fat, such as linoleic, palmitic, stearic, oleic, and myristic oils; 4RF18, Mucedola Srl, Italy) and provided a different kind of drinking water *ad libitum*. Three treatment groups numbered eight males and eight females: SCMmw, BSMmw, and lmw; and three treatment groups numbered nine males and eight females: BCmw, BCPMmw, and mwMix group. The latter group was given a different kind of mineral water each week, in the following order: lmw, BCPMmw, SCMmw, lmw, BSMmw, BCmw, lmw, and BCPMmw. A control group of nine males and seven females was given tap water (tapw). Body weight and blood biochemistry were monitored (see section “Body Weight and Blood Biochemistry Testing” below).

### Body Weight and Blood Biochemistry Testing

Body weight and serum levels of total cholesterol, high-density lipoprotein (HDL) cholesterol, glucose, and triglycerides were measured. All animals were weighed weekly throughout the trial. Blood samples were collected from all animals just before the administration of HCD (T0), and subsequently, at 2 (T2) and 4 (T4) months on HCD (the MetS induction phase), and at 2 months (T6) on LCD (the treatment phase). All mice were fasted for 3 h before blood collection. BD Microtainer tubes (Becton, Dickinson and Company) were used to separate serum from blood. Serum levels of total cholesterol, HDL cholesterol, glucose, and triglycerides were determined by an automatic biochemical analyser (Keylab, BPC BioSed Srl, Rome, Italy).

### Statistical Analysis

Mean values per group and standard deviations (SDs) were used to describe changes in body weight and serum total cholesterol, HDL cholesterol, glucose, and triglycerides during both the MetS induction and treatment phases. Both absolute changes (calculated by subtracting the initial value from the final value) and relative changes (calculated by dividing the absolute change by the initial value) in body weight and blood biochemical parameters were considered for the parametric statistical analysis.

Between-gender analyses were performed by unpaired, two-tailed Student’s *t*-test. Between-group analyses (treatment groups vs. control) were performed by one-way ANOVA to determine the significance of the main effect of treatment, and by two-way ANOVA to determine the significance of the main effects of sex and treatment, as well as of the interaction between them. Dunnett’s test was then performed, wherever applicable, to compare the different group means against that of the (tapw) control group.

GraphPad Prism 6 version 6.07 for Windows, GraphPad Software, La Jolla, CA, United States,^2^ was used for all statistical analyses. Differences were considered significant at *P* < 0.05.

## Results

### The Metabolic Syndrome Induction Phase

Mice of both sexes presented the co-occurrence of obesity, hypercholesterolaemia, and hyperglycaemia at T4, attesting to the development of MetS. Results are shown in Table 2 and described further in the subsections below.

**TABLE 2:** Mean absolute and relative changes in body weight and biochemical parameters among C57BL/6 mice after 4 months on HCD, by gender.

Parameters	HCD		Gender difference *P-*value
	*Female*	*Male*	

	Mean ± SD (*n*)	Mean ± SD (*n*)	
Body weight (BW, g)			
T0 BW	19.42 ± 1.22(55)	22.22 ± 1.78(60)	
T4 BW	32.40 ± 5.25(55)	43.86 ± 4.62(60)	
ΔBW = T4-T0	12.98 ± 5.59(55)	21.64 ± 4.03(60)	< 0.0001****
*C*BW = ΔBW/T0	0.68 ± 0.31(55)	0.98 ± 0.19(60)	< 0.0001****
Total cholesterol (TC, mg/dL)			
T0 TC	102.82 ± 14.28(55)	106.07 ± 20.16(59)	
T4 TC	177.48 ± 39.96(54)	232.64 ± 58.38(59)	
ΔTC = T4-T0	74.31 ± 34.55(54)	126.28 ± 54.34(58)	< 0.0001****
*C*TC = ΔTC/T0	0.72 ± 0.31(54)	1.23 ± 0.64(58)	< 0.0001****
High-density lipoprotein cholesterol (HDL, mg/dL)			
T0 HDL	67.31 ± 11.88(55)	82.64 ± 23.77(59)	
T4 HDL	102.67 ± 18.04(54)	130.61 ± 25.71(59)	
ΔHDL = T4-T0	35.30 ± 24.11(54)	47.34 ± 40.63(58)	0.0615
*C*HDL = ΔHDL/T0	0.59 ± 0.45(54)	0.71 ± 0.65(58)	0.2618
Blood glucose (Glu, mg/dL)			
T0 Glu	145.24 ± 29.57(55)	148.92 ± 33.43(59)	
T4 Glu	162.67 ± 25.95(54)	229.02 ± 50.64(59)	
ΔGlu = T4-T0	16.96 ± 37.78(54)	81.41 ± 55.45(58)	< 0.0001****
*C*Glu = ΔGlu/T0	0.18 ± 0.41(54)	0.63 ± 0.53(58)	< 0.0001****
Triglycerides (TG, mg/dL)			
T0 TG	59.24 ± 17.07(55)	76.27 ± 24.86(59)	
T4 TG	47.19 ± 10.41(54)	53.76 ± 17.65(59)	
ΔTG = T4-T0	−12.26 ± 19.88(54)	−22.83 ± 29.85(58)	0.0307*
*C*TG = ΔTG/T0	−0.13 ± 0.36(54)	−0.19 ± 0.54(58)	0.4938

*HCD, high-calorie diet; SD, standard deviation; n, number of animals; T0, before HCD feeding; T4, after 4 months on HCD. Δ, absolute change; C, relative change; P values were calculated using the unpaired two-tailed Student’s t-test. Asterisks indicate statistical significance.*

**FIGURE 2:**
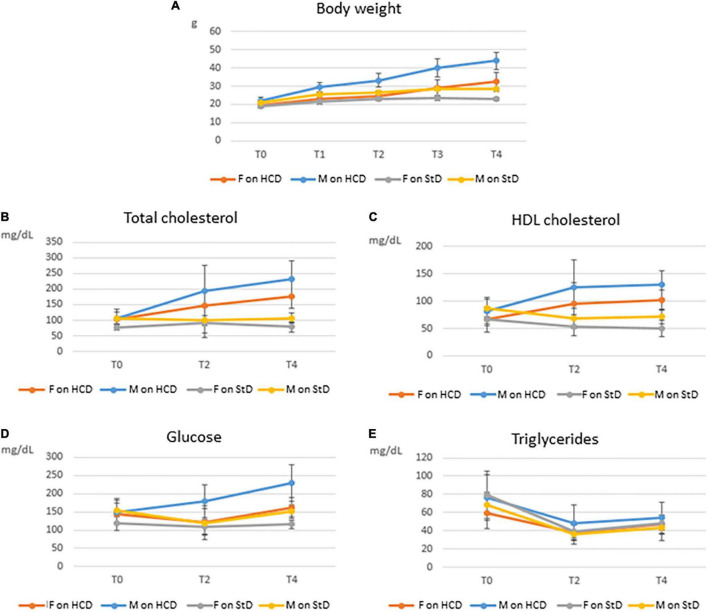
Trends in body weight and blood biochemistry among C57BL/6 mice over 4 months on HCD (the MetS induction phase) or StD, by gender. Means and SDs of body weight **(A)**, total cholesterol **(B)**, high-density lipoprotein (HDL) cholesterol **(C)**, glucose **(D)**, and triglycerides **(E)** for both male (M) and female (F) mice on high-calorie diet (HCD) or standard diet (StD), before (T0) and after 1 (T1), 2 (T2), 3 (T3), and 4 (T4) months feeding on HCD or StD.

Trends in body weight and blood biochemistry are presented in Figure 2. An overall marked increase in all parameters, with the exception of triglycerides, was observed in mice on HCD. This increase was more substantial in males than in females. Changes in body weight and blood biochemistry among healthy untreated mice on StD over the entire 6-month period are shown in Supplementary Table 1. Trends in body weight and blood biochemistry of combined males and females are shown in Supplementary Figure 1.

#### Changes in Body Weight

Both male and female mice gained weight on the HCD, though males did so more rapidly than females over the 4-month period. At T4, the average weight gain of males was 21.64 g and that of females was 12.98 g. Significant differences in both absolute (ΔBW) and relative (*C*BW) body weight gains were found between males and females (*P* < 0.0001) (Table 2). On the other hand, mice on StD (healthy untreated group) gained very little weight over the same period compared with mice on HCD (Figure 2A).

#### Changes in Blood Biochemistry

Total cholesterol rapidly rose in mice on HCD. Here, too, males showed steeper increases than females over the MetS induction phase (Figure 2B). Significant differences in gains in both absolute (ΔTC) and relative (*C*TC) total cholesterol were found between males and females on HCD at T4 (*P* < 0.0001) (Table 2). On the other hand, total cholesterol in healthy mice on StD remained fairly steady over the 4 months in both sexes (Figure 2B),

High-density lipoprotein cholesterol rose rapidly from T0 to T2, and then remained relatively constant until T4 in both sexes on HCD (Figure 2C). Gender differences in both absolute (ΔHDL) and relative (*C*HDL) HDL cholesterol gains were not significant at T4, even though a trend toward statistical significance was observed in ΔHDL (*P* < 0.0615) (Table 2). HDL cholesterol in healthy mice on StD, on the other hand, decreased slightly over the same period in both sexes (Figure 2C).

Glucose gains at T4 – both absolute (ΔGlu) and relative (*C*Glu) – differed significantly between males and females on HCD (*P* < 0.0001) (Table 2). Glucose climbed in males on HCD over the MetS induction phase. In females, however, glucose initially declined (T2) and then gradually rose, ultimately showing a modest increase at T4 as compared to T0 (Figure 2D). A similar trend was observed in healthy males on StD, although no change in glucose levels was observed at T4 as compared to T0. Healthy females on StD, however, maintained the same level of glucose throughout the period (Figure 2D).

In contrast with the other biochemical parameters, triglycerides sharply dropped at T2 and then climbed slightly at T4 in both males and females on HCD and StD alike. Compared to T0, however, a decrease was seen at T4 in all animals (Figure 2E). A significant gender difference was observed in the absolute (ΔTG) drops in the levels of triglycerides (*P* = 0.0307) (Table 2).

### The Treatment Phase

**FIGURE 3:**
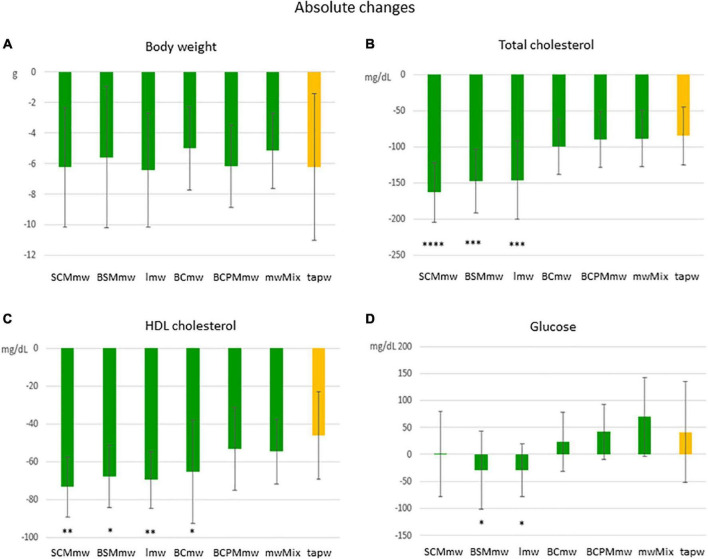
Main effect of treatment on absolute changes in physical and biochemical parameters among treated mice. The figure shows average absolute differences (T6–T4 ± SDs; **A–D**) in physical and biochemical parameters in different mineral water-treated mice and tap water-treated control group. Each group includes both males and females. The groups are: SCMmw, sulphate-, calcium-, and magnesium-rich mineral water; BSMmw, bicarbonate-, sulphate-, and magnesium-rich mineral water; lmw, light mineral water; BCmw, bicarbonate- and calcium-rich mineral water; BCPMmw, bicarbonate-, calcium-, potassium-, and magnesium-rich mineral water; mwMix, alternation of NMWs; tapw, low mineral content tap water. Between-group analysis for the effect of treatment was performed by one-way ANOVA, followed by Dunnett’s test, comparing each group against the control group. *P ≤ 0.05, **P ≤ 0.01, ***P ≤ 0.001, and ****P ≤ 0.0001.

**FIGURE 4:**
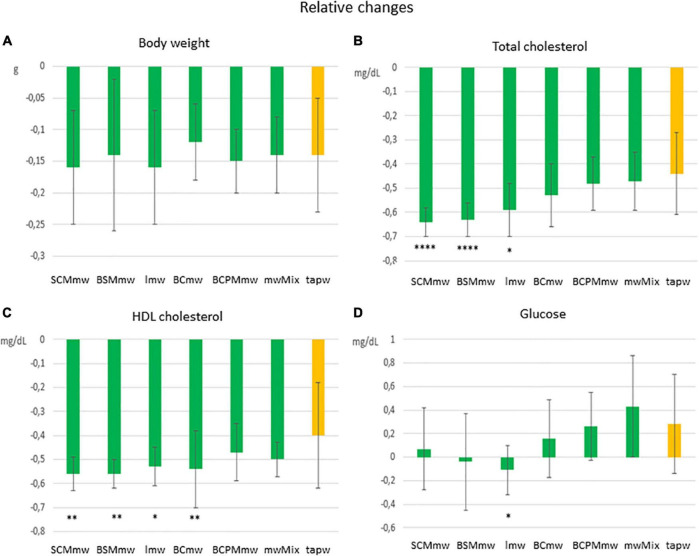
Main effect of treatment on relative changes in physical and biochemical parameters among treated mice. The figure shows average relative differences (T6–T4/T4 ± SDs; **A–D**) in physical and biochemical parameters in different mineral water-treated mice and tap water-treated control group. Each group includes both males and females. The groups are: SCMmw, sulphate-, calcium-, and magnesium-rich mineral water; BSMmw, bicarbonate-, sulphate-, and magnesium-rich mineral water; lmw, light mineral water; BCmw, bicarbonate- and calcium-rich mineral water; BCPMmw, bicarbonate-, calcium-, potassium-, and magnesium-rich mineral water; mwMix, alternation of NMWs; tapw, low mineral content tap water. Between-group analysis for the effect of treatment was performed by one-way ANOVA, followed by Dunnett’s test, comparing each group against the control group. *P ≤ 0.05, **P ≤ 0.01, and ****P ≤ 0.0001.

**FIGURE 5:**
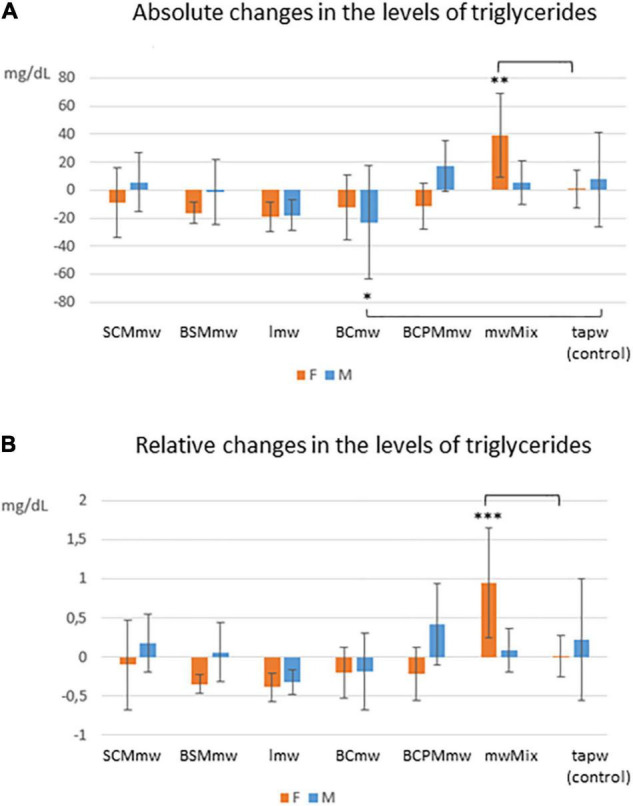
Interaction effect of sex and treatment on triglycerides among treated mice. The figure shows average absolute (T6–T4; **A**) and relative (T6–T4/T4; **B**) differences ± SDs in triglyceride levels among mineral water-treated mice and tap water-treated controls. The groups are: SCMmw, sulphate-, calcium-, and magnesium-rich mineral water; BSMmw, bicarbonate-, sulphate-, and magnesium-rich mineral water; lmw, light mineral water; BCmw, bicarbonate- and calcium-rich mineral water; BCPMmw, bicarbonate-, calcium-, potassium-, and magnesium-rich mineral water; mwMix, alternation of NMWs; tapw, low mineral content tap water. Between-group analysis by two-way ANOVA was performed to determine the effects of sex, treatment, and the interaction between the two, followed by Dunnett’s test comparing each group against the control group. *P ≤ 0.05, **P ≤ 0.01, and ***P ≤ 0.001.

As of T4, mice were fed an LCD and treated with either NMW (mineral water-treated mice) or tap water (tap water-treated mice or control group) for 2 months. Body weight and serum levels of total cholesterol, HDL cholesterol, glucose and triglycerides were measured at the end of the 2-month treatment phase (T6). Results are shown in Supplementary Table 2, Tables 3, 4, and Figures 3–5.

**TABLE 3:** Absolute and relative changes of body weight and blood biochemical parameters of treated mice, by treatment group and gender.

	Mineral water-treated mice					
Parameters	SCMmw		BSMmw		lmw	
	
	Female (*n*)	Male (*n*)	Female (*n*)	Male (*n*)	Female (*n*)	Male (*n*)

	mean ± SD					
Body weight (BW, g)						
ΔBW = T6-T4	−4.67 ± 4.24(7)	−7.62 ± 3.19(8)	−4.57 ± 6.23(8)	−6.60 ± 2.16(8)	−6.49 ± 5.20(8)	−6.39 ± 1.64(8)
*C*BW = ΔBW/T4	−0.14 ± 0.12(7)	−0.18 ± 0.06(8)	−0.13 ± 0.17(8)	−0.16 ± 0.04(8)	−0.18 ± 0.12(8)	−0.15 ± 0.04(8)
Total cholesterol (TC, mg/dL)						
ΔTC = T6-T4	−139.43 ± 25.55(7)	−186.86 ± 41.93(7)	−129.50 ± 38.24(8)	−164.50 ± 44.46(8)	−108.00 ± 44.28(8)	−184.50 ± 31.16(8)
*C*TC = ΔTC/T4	−0.64 ± 0.05(7)	−0.65 ± 0.08(7)	−0.62 ± 0.07(8)	−0.64 ± 0.07(8)	−0.53 ± 0.12(8)	−0.64 ± 0.05(8)
High-density lipoprotein cholesterol (HDL, mg/dL)						
ΔHDL = T6-T4	−69.71 ± 18.60(7)	−77.14 ± 12.59(7)	−62.00 ± 16.56(8)	−74.00 ± 15.12(8)	−63.00 ± 15.96(8)	−76.50 ± 10.57(8)
*C*HDL = ΔHDL/T4	−0.58 ± 0.07(7)	−0.55 ± 0.07(7)	−0.57 ± 0.06(8)	−0.55 ± 0.06(8)	−0.55 ± 0.10(8)	−0.52 ± 0.06(8)
Blood glucose (Glu, mg/dL)						
ΔGlu = T6-T4	33.14 ± 42.69(7)	−32.00 ± 95.41(7)	14.00 ± 53.41(8)	−73.00 ± 63.96(8)	−12.50 ± 41.48(8)	−45.00 ± 52.58(8)
*C*Glu = ΔGlu/T4	0.24 ± 0.29(7)	−0.09 ± 0.34(7)	0.18 ± 0.46(8)	−0.26 ± 0.20(8)	−0.06 ± 0.25(8)	−0.16 ± 0.17(8)
Triglycerides (TG, mg/dL)						
ΔTG = T6-T4	−9.14 ± 25.16(7)	5.71 ± 21.02(7)	−16.25 ± 7.89(8)	−1.50 ± 23.51(8)	−19.00 ± 10.42(8)	−18.00 ± 10.90(8)
*C*TG = ΔTG/T4	−0.10 ± 0.57(7)	0.18 ± 0.37(7)	−0.35 ± 0.12(8)	0.06 ± 0.38(8)	−0.39 ± 0.18(8)	−0.32 ± 0.16(8)

	**Mineral water-treated mice**					
	
**Parameters**	**BCmw**		**BCPMmw**		**mwMix**	
	
	**Female (*n*)**	**Male (*n*)**	**Female (*n*)**	**Male (*n*)**	**Female (*n*)**	**Male (*n*)**

	**Mean ± SD**					

Body weight (BW, g)						
ΔBW = T6-T4	−3.36 ± 2.73(8)	−6.48 ± 1.75(9)	−4.53 ± 2.59(8)	−7.66 ± 1.88(9)	−5.50 ± 2.28(8)	−5.40 ± 2.72(9)
*C*BW = ΔBW/T4	−0.10 ± 0.007(8)	−0.14 ± 0.04(9)	−0.13 ± 0.06(8)	−0.17 ± 0.04(9)	−0.15 ± 0.05(8)	−0.12 ± 0.06(9)
Total cholesterol (TC, mg/dL)						
ΔTC = T6-T4	−90.50 ± 18.26(8)	−108.00 ± 50.04(9)	−76.57 ± 16.07(7)	−100.00 ± 48.11(9)	−58.50 ± 23.61(8)	−115.33 ± 28.67(9)
*C*TC = ΔTC/T4	−0.56 ± 0.08(8)	−0.50 ± 0.16(9)	−0.50 ± 0.06(7)	−0.46 ± 0.14(9)	−0.39 ± 0.13(8)	−0.54 ± 0.06(9)
High-density lipoprotein cholesterol (HDL, mg/dL)						
ΔHDL = T6-T4	−60.00 ± 15.42(8)	−70.22 ± 34.93(9)	−49.71 ± 11.97(7)	−56.00 ± 27.51(9)	−43.00 ± 12.96(8)	−65.11 ± 13.75(9)
*C*HDL = ΔHDL/T4	−0.58 ± 0.09(8)	−0.50 ± 0.21(9)	−0.52 ± 0.06(7)	−0.43 ± 0.14(9)	−0.48 ± 0.09(8)	−0.52 ± 0.05(9)
Blood glucose (Glu, mg/dL)						
ΔGlu = T6-T4	34.00 ± 59.14(8)	14.67 ± 52.88(9)	54.29 ± 41.43(7)	32.00 ± 57.69(9)	95.00 ± 59.78(8)	46.67 ± 80.35(9)
*C*Glu = ΔGlu/T4	0.22 ± 0.40(8)	0.10 ± 0.27(9)	0.37 ± 0.29(7)	0.17 ± 0.26(9)	0.61 ± 0.39(8)	0.27 ± 0.42(9)
Triglycerides (TG, mg/dL)						
ΔTG = T6-T4	−12.50 ± 23.07(8)	−23.11 ± 40.54(9)	−11.43 ± 16.56(7)	17.33 ± 18.19(9)	39.00 ± 29.76(8)	5.33 ± 15.62(9)
*C*TG = ΔTG/T4	−0.20 ± 0.33(8)	−0.19 ± 0.49(9)	−0.22 ± 0.34(7)	0.42 ± 0.52(9)	0.95 ± 0.70(8)	0.09 ± 0.28(9)

	**Tap water-treated mice (control group)**					
	
**Parameters**	**tapw**		***P*-value**			
		
	**Female (*n*)**	**Male (*n*)**	**Sex (S)**	**Treatment (T)**	**Interaction (S × T)**	

	**Mean ± SD**					

Body weight (BW, g)						
ΔBW = T6-T4	−5.32 ± 2.63(7)	−6.92 ± 6.05(9)	0.0084**	0.9077	0.6954	
*C*BW = ΔBW/T4	−0.14 ± 0.07(7)	−0.14 ± 0.11(9)	0.4136	0.7173	0.7739	
Total cholesterol (TC, mg/dL)						
ΔTC = T6-T4	−67.14 ± 25.19(7)	−98.67 ± 44.86(9)	<0.0001****	<0.0001****	0.2895	
*C*TC = ΔTC/T4	−0.44 ± 0.15(7)	−0.44 ± 0.19(9)	0.2066	<0.0001****	0.0998	
High-density lipoprotein cholesterol (HDL, mg/dL)						
ΔHDL = T6-T4	−39.57 ± 12.49(7)	−51.11 ± 28.69(9)	0.0019**	0.0004***	0.9465	
*C*HDL = ΔHDL/T4	−0.44 ± 0.14(7)	−0.36 ± 0.27(9)	0.0878	0.0045**	0.7895	
Blood glucose (Glu, mg/dL)						
ΔGlu = T6-T4	71.00 ± 61.93(7)	18.67 ± 111.12(9)	0.0003***	<0.0001****	0.7745	
*C*Glu = ΔGlu/T4	0.44 ± 0.42(7)	0.15 ± 0.27(9)	<0.0001****	<0.0001****	0.7472	
Triglycerides (TG, mg/dL)						
ΔTG = T6-T4	0.43 ± 13.46(7)	7.56 ± 33.73(9)	0.4692	<0.0001****	0.0078**	
*C*TG = ΔTG/T4	0.01 ± 0.27(7)	0.22 ± 0.78(9)	0.1982	<0.0001****	0.0004***	

*SCMmw, sulphate-, calcium-, and magnesium-rich mineral water; BSMmw, bicarbonate-, sulphate-, and magnesium-rich mineral water; lmw, light mineral water; BCmw, bicarbonate- and calcium-rich mineral water; BCPMmw, bicarbonate-, calcium-, potassium-, and magnesium-rich mineral water; mwMix, alternation of natural mineral waters; tapw, low mineral content tap water; n, number of animals; SD, standard deviation; T4, before any treatment; T6, after 2 months of treatment; Δ, absolute change; C, relative change.*

*P-values were calculated using two-way ANOVA followed by Dunnett’s multiple comparisons test, where each group was compared to the control group of tap water-treated mice. Statistical analysis was performed for the effects of sex (S), treatment (T) and the interaction between the two (S × T) calculated for absolute as well as for relative values. Asterisks indicate statistical significance.*

**TABLE 4:** Main effect of gender on absolute and relative changes in body weight and blood biochemistry among treated mice.

Parameters	Female (*n*)	Male (*n*)	*P*-value

	Mean ± SD		
Body weight (BW, g)			
ΔBW = T6-T4	−4.92 ± 3.86(54)	−6.72 ± 3.08(60)	0.0067**
*C*BW = ΔBW/T4	−0.14 ± 0.10(54)	−0.15 ± 0.06(60)	0.5139
Total cholesterol (TC, mg/dL)			
ΔTC = T6-T4	−95.74 ± 39.90(53)	−133.86 ± 54.25(59)	< 0.0001****
*C*TC = ΔTC/T4	−0.52 ± 0.13(53)	−0.55 ± 0.14(59)	0.2441
High-density lipoprotein cholesterol (HDL, mg/dL)			
ΔHDL = T6-T4	−55.42 ± 17.56(53)	−66.54 ± 23.69(59)	0.0061**
*C*HDL = ΔHDL/T4	−0.53 ± 0.10(53)	−0.49 ± 0.16(59)	0.1203
Blood glucose (Glu, mg/dL)			
ΔGlu = T6-T4	40.62 ± 59.83(53)	−2.71 ± 83.20(59)	0.0022**
*C*Glu = ΔGlu/T4	0.28 ± 0.40(53)	0.04 ± 0.34(59)	0.0008***
Triglycerides (TG, mg/dL)			
ΔTG = T6-T4	−3.98 ± 26.58(53)	−0.88 ± 27.94(59)	0.5498
*C*TG = ΔTG/T4	−0.04 ± 0.58(53)	−0.07 ± 0.51(59)	0.2879

*SD, standard deviation; n, number of animals; Δ, absolute change; C, relative change. P-values were calculated using the unpaired two-tailed Student’s t-test for absolute as well as relative changes. Asterisks indicate statistical significance.*

An overall rapid fall in both body weight and levels of total and HDL cholesterol were observed in mice on LCD at T6 (Figures 3, 4). Conversely, an additional increase in blood glucose was recorded at T6 in both mineral water-treated and control mice, with some exceptions (Figures 3, 4). Triglycerides exhibited a mixed trend: increasing in some groups, and stable in others (Figure 5).

The interaction between sex and treatment was significant only for triglycerides. Sex differences were observed in all parameters with the exception of triglycerides, and treatment differences were observed in all parameters except body weight. Further details are given in the subsections below.

#### Body Weight

Mice of all groups lost weight on LCD. The interaction between sex and treatment was not significant (Table 3). Sex was associated with weight loss, but treatment was not. Males lost body weight more rapidly than females over the 2 months of treatment (*P* = 0.0067 in *t*-test for absolute changes, ΔBW; Table 4), whereas no absolute (Figure 3A) or relative (Figure 4A) differences were found between mineral water-treated mice (treated groups, green bars) and tap water-treated mice (control group, yellow bar).

#### Changes in Blood Biochemistry

##### Cholesterol

Serum levels of both total cholesterol and HDL cholesterol decreased in all mice on LCD (Table 3). The interaction between sex and treatment was not significant for either HDL or total cholesterol, while the main effects of sex and treatment were significant for both (Table 3). Total and HDL cholesterol alike dropped more sharply in males than females (*P* < 0.0001 and *P* = 0.0061 in *t*-test for absolute changes of total cholesterol, and HDL cholesterol, respectively; Table 4). Treatment was significantly associated with both absolute and relative changes for these two parameters. Treatment with SCMmw, BSMmw, or lmw substantially reduced the levels of total serum cholesterol of obese mice as compared to tap water (Figures 3B, 4B). These three kinds of NMW as well as BCmw were also able to considerably reduce the levels of serum HDL cholesterol in obese mice (Figures 3C, 4C).

##### Blood Glucose

By T6, glycaemia worsened in the BCmw, BCPMmw, mwMix, and tapw groups (both males and females), but improved among males on SCMmw and BSMmw, and in both males and females on lmw (Table 3). Here too, as with total and HDL cholesterol levels, the interaction between sex and treatment was not significant (Table 3). On the other hand, sex and treatment were significantly associated with both absolute (ΔGlu) and relative (*C*Glu) changes in glucose levels (Table 3). A significant and considerable overall increase in glycaemia in females and a slight decrease in glycaemia in males were observed (*P* = 0.0022 for ΔGlu and *P* = 0.008 for *C*Glu in *t*-test; Table 4). A reduction in blood glucose levels following treatment was observed in the BSMmw and lmw groups only when absolute changes were considered, and in the lmw group only when relative changes were examined (Figures 3D, 4D).

##### Triglycerides

A mixed trend was observed for triglycerides at T6, characterised by between-genders and between-group differences. In some groups, the level of triglycerides declined, in some it rose, and in others it remained constant (Table 3). The interaction between sex and treatment was significant for both absolute (ΔTG) and relative (*C*TG) changes (Table 3). Significant differences were observed when analysing triglyceride levels at T6: while the levels of triglycerides decreased in males on BCmw (absolute changes) (Figure 5A), it increased among females on mwMix, both in terms of absolute and relative changes (Figures 5A,B).

## Discussion

The prevalence of MetS, a cluster of cardiovascular risk factors, such as impaired glucose homoeostasis, dyslipidaemia, obesity, and hypertension, is increasing worldwide (23). The main aim of our study was to investigate the extent to which the administration of NMW of different kinds, in combination with an LCD, may affect different features of MetS, such as body weight, total and HDL cholesterol, glucose, and triglyceride levels. The study was performed on C57BL/6 mice, as rodent models are known to be suitable substitutes for humans to study obesity and its related biomarkers. And yet, to the best of our knowledge, this is the first study on the metabolic impact of NMWs in an animal model of MetS to be published thus far. We considered both absolute and relative changes in each analysed parameter in order to better describe the real magnitude of the observed differences between gender and treatment groups.

After 2 months on LCD, the most effective mineral waters in reducing the blood levels total cholesterol, HDL cholesterol, and glucose, were the those rich in bicarbonate, sulphate, calcium, and magnesium (SCMmw and BSMmw), and the minimally mineralised water (lmw).

High-fat diets have been used to model obesity, dyslipidaemia, and insulin resistance in rodents for many decades. An HFD causes several complications, such as cardiac hypertrophy, myocardial necrosis, fatty liver disease (hepatic steatosis), and renal dysfunction (24). The addition of carbohydrates to an HFD increases body weight, abdominal fat deposition, hyperinsulinaemia, hyperglycaemia, and hyperleptinaemia in mice, who thus develop all of the complications present in human MetS (24, 25). A diet high both in fat and carbohydrates closely mimics the modern Western diet. Fructose consumption is associated with metabolic changes similar to those observed in MetS (26, 27). Studies have shown fructose-feeding to be superior to glucose or starch for the induction of MetS in animal models (28), and the administration of HCD with fructose-enriched drinking water to induce MetS in rodents (29, 30). We therefore administered an HCD consisting of an HFD (60% energy from fat) and 10% fructose-enriched drinking water *ad libitum* to C57BL/6 mice during the MetS phase.

The wild-type C57BL/6 mouse is particularly susceptible to weight gain when fed HFD compared to other genetically manipulated mouse models (31). Over time, C57BL/6 mice typically exhibit features commonly associated with MetS in humans, which include obesity, insulin resistance, glucose intolerance, hyperlipidaemia, hypertriglyceridaemia, and hypertension (32, 33). Having said that, no animal model has thus far been developed, that can faithfully reproduce human obesity.

In this study, C57BL/6 mice of both sexes developed obesity, hypercholesterolaemia (total, as well as HDL cholesterol), and hyperglycaemia after 4 months on HCD. High levels of both total cholesterol and HDL cholesterol are usual in obese rodent models (34, 35), whereas human MetS, is characterised by high levels of total cholesterol but reduced levels of HDL cholesterol (1). The reason rats and mice exhibit high levels of HDL cholesterol is that they are naturally deficient in the cholesteryl ester transfer protein (CETP) (36). This protein plays a significant role in the remodelling of plasma HDL cholesterol by promoting the passive exchange of neutral lipids between plasma lipoproteins (36), and its absence explains the increase in the levels of HDL cholesterol in mice on HCD.

Metabolic syndrome is associated with greater production of atherogenic lipoprotein particles, such as LDL cholesterol. Elevated levels of LDL cholesterol have proven to be directly associated with the risk of atherosclerotic cardiovascular events, including heart attack and stroke. HDL cholesterol, the “good cholesterol,” on the other hand, metabolises lipid hydroperoxides and thus prevents the oxidation of LDL cholesterol and its atherogenic structural modification. In short, HDL cholesterol acts as an antioxidant and antagonises atherosclerosis, by preventing inflammation and oxidative stress (37).

High level of triglycerides and low levels of HDL cholesterol commonly characterise dyslipidemia in human obesity. It has been observed that, not only levels of HDL cholesterol are altered in obesity, but also its distribution and metabolism, which often lead to a dysfunction of HDL particles (38), which may explain the higher risk of developing atherosclerosis in obesity.

It is widely accepted that male rodents tend to gain more fat and body weight than females on HCD. For this reason, studies based on *in vivo* modelling of MetS usually use male mice and rats (18). C57BL/6 is an obesity-prone mouse strain in which HFD-induced obesity is influenced by the sex and age of the animal (39). We included both sexes in our study to better characterise the features of MetS by gender in this strain. Sex differences emerged, as expected, during the MetS induction phase. Both absolute and relative gains in body weight, total cholesterol, and glucose in males were greater than those observed in females on HCD, in agreement with previous studies (34). No sex differences in blood levels of HDL cholesterol were found, however. This is at odds with the study published by Ding et al. (40), in which a significant gender difference is described. Differences in the type and duration of HCD administration, as well as in the age of the mice used in the study, may explain the discrepancy between our findings and the literature.

The observed trend in triglyceride levels among mice on HCD was different from what we had expected: a decline was recorded in both sexes, which was more pronounced in males. This finding is in contrast with previous studies showing either relatively stable serum triglyceride concentrations (19, 25, 34) or increased concentrations in both sexes of C57BL/6 mice on HFD, with gender differences (40). Again, the discrepancy between our findings and the literature may be due to differences in study design. Better standardisation in future study designs may help clarify some of these inconsistencies.

Sex differences appeared during the treatment phase as well. Body weight, total cholesterol, and HDL cholesterol decreased in treated mice of both sexes on LCD, though the absolute reductions were more marked in males than in females. These sex differences disappeared when relative changes were considered. Glucose, on the other hand, decreased in some treated groups on LCD and increased in others (control group included) with gender differences. An overall increase in glycaemia in females and a slight decrease in glycaemia in males was observed. Conceivably, different endocrine signals are involved in male and female mice. To gain a better understanding of the effects of 2 months on a LCD on blood glucose levels, glucose-insulin homoeostasis will be investigated in a future study. However, the fact that no interaction between sex and treatment was recorded for any of these parameters, suggests that the changes in total cholesterol, HDL cholesterol, and glucose levels observed in females and males in the treatment group were slight and not significant. In other words, females in the group responded to the treatment in the same way as males as far as these parameters are concerned.

Conversely, triglycerides showed a significant sex-treatment interaction. This result indicates that the changes in triglyceride levels following treatment were different between males and females. Decreases, increases, or minor changes in triglyceride levels were indeed observed between males and females (within groups) and between groups (treatment vs. control). Ultimately, our findings confirm C57BL/6 males on HCD to be more inclined to develop MetS features than females, but only as concerns obesity, hypercholesterolaemia, and hyperglycaemia. Similarly, males with MetS fed LCD and treated with a NMW tended to lose body weight, and reduce total cholesterol and HDL cholesterol more than females. Females responded significantly to the treatment as well, albeit less vigorously. These results suggest that obese C57BL/6 mice of both sexes may be used to investigate the effects of treatment on MetS.

Compared to tap water, the NMW treatments affected all of the studied MetS features, with the exception of body weight. While SCMmw, BSMmw, and lmw significantly reduced both total and HDL cholesterol, the latter two – BSMmw and lmw – also reduced blood glucose levels. BCmw only reduced HDL cholesterol levels. In short, BSMmw and lmw appeared to be the most effective in mitigating MetS features by simultaneously acting on total cholesterol, HDL cholesterol, and glucose levels. However, for a more complete understanding of the effects of NMWs on MetS, additional parameters, besides body weight, lipid profile, and blood sugar, should be evaluated, such as fat mass, inflammation, insulin resistance, and liver health.

To date, few studies have been conducted to investigate the efficacy of NMWs in mitigating MetS features in humans, and these have yielded inconsistent results. Some studies showed no effects on total, LDL, HDL cholesterol, and triglycerides in hyperlipidaemic subjects treated with a bicarbonate- and calcium-rich water (41) or in moderately hypercholesterolaemic and overweight subjects treated with a sulphate-, magnesium-, sodium-, chloride-, and bicarbonate-rich water (42). Similarly, no effect on glucose levels was observed in moderately hypercholesterolaemic subjects treated with a bicarbonate-, sodium-, and chloride-rich water (43). Others documented a reduction in total and LDL cholesterol and glucose, as well as an increase in HDL cholesterol among moderately hypercholesterolaemic and overweight subjects treated with a bicarbonate-, sodium-, and chloride-rich water (44–46). However, variability between studies in terms of population size, the characteristics of subjects enrolled and the type of mineral water administered, make it difficult to draw definitive conclusions on the potential role of NMWs in the modulation of MetS features.

Natural mineral waters have documented beneficial physiological effects on human health. Several studies have demonstrated the positive effects of bicarbonate-rich mineral waters on the digestive tract (13), on cardiometabolic risk biomarkers and in the prevention of cardiovascular diseases (46). Sulphate-rich mineral waters, characterised by the presence of sulphate anions, have numerous beneficial properties, including the promotion of intestinal peristalsis. Sulphate, in combination with magnesium, has proven to be highly efficacious in the treatment of functional constipation (14, 47). Magnesium promotes lipid metabolism and offers protection from cardiovascular disease (48). Mineral waters characterised by a more complex composition, such as sulphate-, bicarbonate-, calcium-, and magnesium-rich waters, has shown therapeutic properties in functional disorders of the biliary tract (15). On the other hand, mineral waters with a very low mineral content have a diuretic effect and are indicated in cases of urinary stones, as they facilitate uric acid clearance (7). All of these documented beneficial properties of specific minerals in drinking waters may explain our finding, that SCMmw, BSMmw, and lmw were the most efficacious in reducing total and HDL cholesterol and glucose, likely by promoting bowel movements, lipid metabolism, and diuresis.

## Conclusion

In conclusion, this study was designed to shed light on: (i) the appropriateness of using C57BL/6 mice of both sexes as animal models of MetS, and (ii) the effect of NMWs on biochemical indicators associated with MetS. Our findings suggest that C57BL/6 mice of both sexes may be included in MetS research, even though males responded to both the induction and treatment of MetS more vigorously than females. In addition, the administration of some mineral waters – such as those rich in sulphate, magnesium, and bicarbonate, or those minimally mineralised – coupled with an LCD may positively affect MetS features and may therefore provide a valuable addition to the standard MetS treatment. Further studies are needed to confirm these results and to extend them to humans.

## Data Availability Statement

The original contributions presented in the study are included in the article/Supplementary Material, further inquiries can be directed to the corresponding author.

## Ethics Statement

The animal study was reviewed and approved by the Italian Ministry of Health (Permit number 938/2018-PR-14/12/2018).

## Author Contributions

CM conceived, designed, and coordinated the study, and wrote the manuscript. LN, AM, FT, PF, RB, and CM conducted the experiments. CM and FC performed the statistical analysis. LN and CM participated in the presentation of the published work. All authors reviewed, read, and approved the submitted version.

## Conflict of Interest

The authors declare that the research was conducted in the absence of any commercial or financial relationships that could be construed as a potential conflict of interest.

## Publisher’s Note

All claims expressed in this article are solely those of the authors and do not necessarily represent those of their affiliated organizations, or those of the publisher, the editors and the reviewers. Any product that may be evaluated in this article, or claim that may be made by its manufacturer, is not guaranteed or endorsed by the publisher.

## Footnotes

1
https://www.gruppo.acea.it/al-servizio-delle-persone/acqua/acea-ato-2/la-qualita-della-tua-acqua

2
www.graphpad.com
