# Supplementary material for: Natural Mineral Waters and Metabolic Syndrome: Insights From Obese Male and Female C57BL/6 Mice on Caloric Restriction
Source: Front Nutr. 2022 May 24;9:886078. doi: 10.3389/fnut.2022.886078 (PMC9172593; doi:10.3389/fnut.2022.886078)
Supplement: Supplementary file 1 [file Data_Sheet_1.docx]

| Parameters | Untreated mice on StD  Female Male | | Gender difference  *P* value |
| --- | --- | --- | --- |
|  | **mean ± SD (n)** | **mean ± SD (n)** |  |
| Body weight (BW, g)  T0 BW  T6 BW  ΔBW = T6 -T0  *C*BW = ΔBW/T0  Total cholesterol (TC, mg/dL)  T0 TC  T6 TC  ΔTC = T6-T0  *C*TC = ΔTC/T0  High-density lipoprotein cholesterol (HDL, mg/dL)  T0 HDL  T6 HDL  ΔHDL = T6-T0  *C*HDL = ΔHDL/T0  Blood glucose (Glu, mg/dL)  T0 Glu  T6 Glu  ΔGlu = T6-T0  *C*Glu = ΔGlu/T0  Triglycerides (TG, mg/dL)  T0 TG  T6 TG  ΔTG = T6-T0  *C*TG = ΔTG/T0 | 18.87 ± 0.92 (5)  24.96 ± 0.65 (5)  6.09 ± 1.13 (5)  0.33 ± 0.08 (5)    77.60 ± 10.81 (5)  59.20 ± 7.69 (5)  -18.40 ± 4.56 (5)  -0.24 ± 0.04 (5)    66.40 ± 23.56 (5)  41.60 ± 6.07 (5)  -24.80 ± 24.85 (5)  -0.29 ± 0.31 (5)    118.80 ± 18.85 (5)  136.00 ± 32.50 (5)  17.20 ± 30.33 (5)  0.20 ± 0.49 (5)  79.40 ± 26.55 (5)  32.40 ± 5.73 (5)  -47.00 ± 22.91 (5)  -0.55 ± 0.16 (5) | 21.07 ± 0.55 (5)  31.07 ± 2.27 (5)  10.00 ± 1.87 (5)  0.47 ± 0.08 (5)  105.20 ± 28.83 (5)  72.80 ± 6.57 (5)  -32.40 ± 30.41 (5)  -0.27 ± 0.21 (5)  87.80 ± 15.21 (5)  54.40 ± 6.07 (5)  -33.40 ± 15.39 (5)  -0.37 ± 0.12 (5)  154.00 ± 32.74 (5)  243.20 ± 43.58 (5)  89.20 ± 45.53 (5)  0.61 ± 0.35 (5)  68.00 ± 14.51 (5)  43.20 ± 11.45 (5)  -24.80 ± 11.03 (5)  -0.36 ± 0.12 (5) | 0.0039 **  0.0244 *  0.3385  0.7617  0.5291  0.6051  0.0410 *  0.1664  0.0867  0.0664 |

**Table S1.** **Mean** **absolute and relative changes in body weight and biochemical parameters among healthy C57BL/6 mice after six months on StD, by gender**

StD, standard diet; SD, standard deviation; n, number of animals; T0, before StD feeding; T6, after six months on StD; Δ, absolute change; *C*, relative change; *P* values were calculated using the unpaired two-tailed Student’s t-test. Asterisks indicate statistical significance.

**
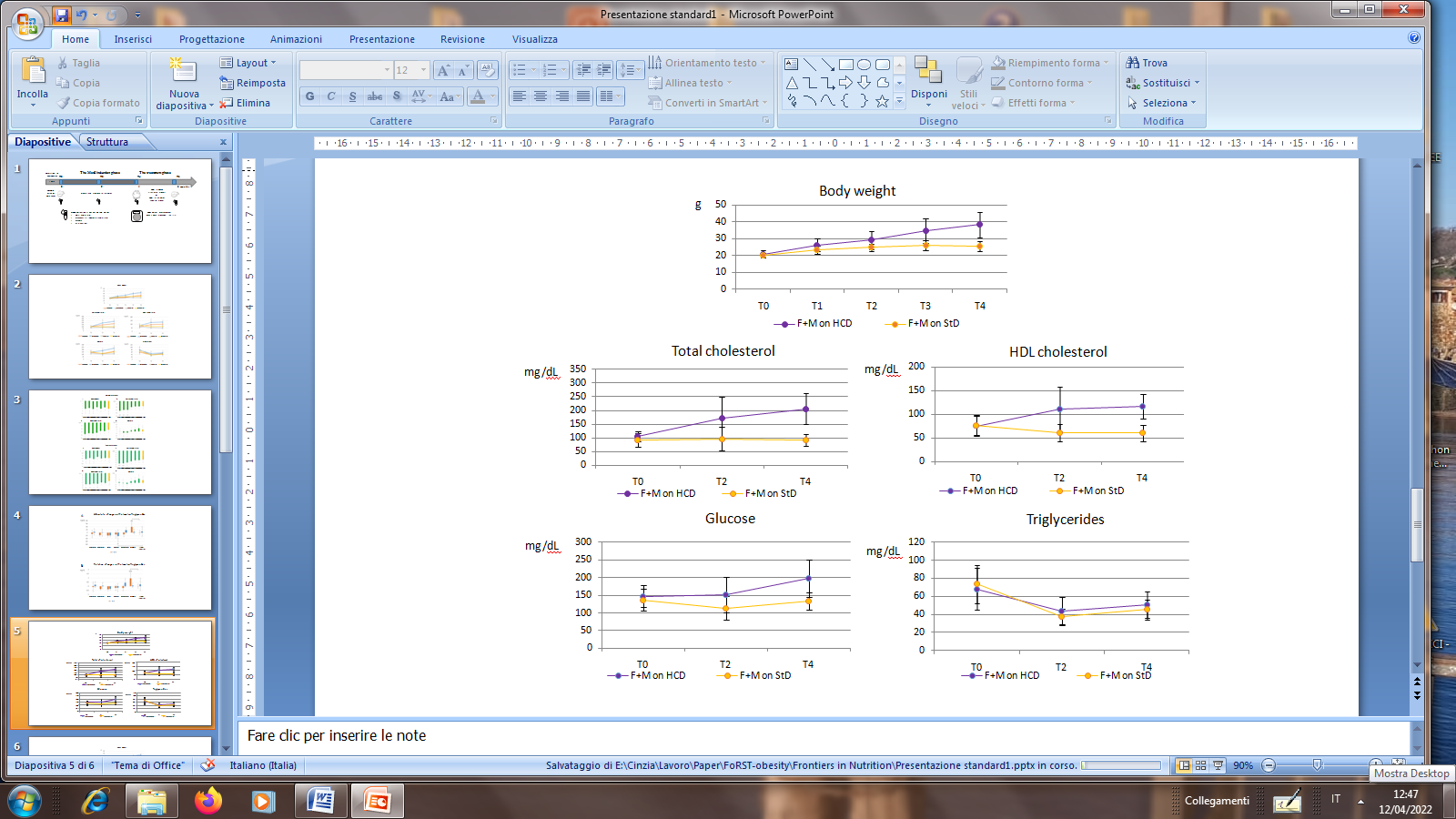
**

**Figure S1. Trends in body weight and blood biochemistry among C57BL/6 mice, which include both males and females, over four months on HCD (the MetS induction phase) or StD.** Means and standard deviations of body weight, total cholesterol, high-density lipoprotein (HDL) cholesterol, glucose and triglycerides for both male and female (F+M) mice on high calorie diet (HCD) or standard diet (StD), before (T0) and after one (T1), two (T2), three (T3) and four (T4) months feeding on HCD or StD.

**Table 2S.** C**hanges of body weight and blood biochemical parameters of treated mice, by treatment group and gender.**

| **Parameters** | **Mineral water-treated mice**  **SCMmw BSMmw lmw**  **Female (n) Male (n) Female (n) Male (n) Female (n) Male (n)** | | | | | |
| --- | --- | --- | --- | --- | --- | --- |
|  | **mean ± SD** | | | | | |
| **Body weight (BW, g)**  **T4 BW**  **T6 BW**  **Total cholesterol (TC, mg/dL)**  **T4 TC**  **T6 TC**  **High-density lipoprotein cholesterol (HDL, mg/dL)**  **T4 HDLC**  **T6 HDLC**  **Blood glucose (Glu, mg/dL)**  **T4 Glu**  **T6 Glu**  **Triglycerides (TG, mg/dL)**  **T4 TG**  **T6 TG** | 31.63 ± 4.27 (8)  26.51 ± 0.73 (7)      223.50 ± 28.08 (8)  80.57 ± 9.64 (7)  119.00 ± 18.24 (8)  49.14 ± 4.45 (7)    161.00 ± 23.40 (8)  188.57 ± 32.86 (7)  46.50 ± 13.17 (8)  39.43 ± 16.88 (7) | 40.33 ± 5.16 (8)  32.71 ± 2.29 (8)  285.14 ± 31.81 (7)  99.00 ± 11.66 (8)  140.57 ± 7.81 (7)  63.00 ± 7.33 (8)    235.43 ± 56.49 (7)  203.00 ± 54.88 (8)  58.29 ± 18.16 (7)  63.50 ± 8.67 (8) | 29.46 ± 6.03 (8)  24.89 ± 1.40 (8)    207.50 ± 46.80 (8)  78.00 ± 18.39 (8)    107.50 ± 21.85 (8)  45.50 ± 8.26 (8)    148.00 ± 35.39 (8)  162.00 ± 30.84 (8)    44.50 ± 7.540 (8)  28.25 ± 3.45 (8) | 40.07 ± 4.43 (8)  33.47 ± 2.49 (8)    253.50 ± 43.45 (8)  89.00 ± 7.63 (8)    132.50 ± 15.33 (8)  58.50 ± 5.63 (8)    244.50 ± 68.17 (8)  171.50 ± 24.61 (8)    50.50 ± 22.52 (8)  49.00 ± 15.38 (8) | 32.39 ± 6.85 (8)  25.90 ± 1.99 (8)    195.00 ± 40.16 (8)  87.00 ± 7.93 (8)  114.00 ± 14.81 (8)  51.00 ± 8.75 (8)  176.00 ± 23.13 (8)  163.50 ± 33.36 (8)  49.00 ± 7.01 (8)  30.00 ± 11.51 (8) | 42.51 ± 4.59 (8)  36.12 ± 4.22 (8)    285.50 ± 38.66 (8)  101.00 ± 16.11 (8)    148.00 ± 8.00 (8)  71.50 ± 6.91 (8)  259.00 ± 46.54 (8)  214.00 ± 35.97 (8)    54.00 ± 9.56 (8)  36.00 ± 8.28 (8) |

SCMmw, sulphate-, calcium- and magnesium-rich mineral water; BSMmw, bicarbonate-, sulphate- and magnesium-rich mineral water; lmw, light mineral water; n, number of animals; SD, standard deviation; T4, before any treatment; T6, after two months of treatment.

**Table 2S.** *Cont.*

| **Parameters** | **Mineral water-treated mice**  **BCmw BCPMmw mwMix**  **Female (n) Male (n) Female (n) Male (n) Female (n) Male (n)** | | | | | |
| --- | --- | --- | --- | --- | --- | --- |
|  | **mean ± SD** | | | | | |
| **Body weight (BW, g)**  **T4 BW**  **T6 BW**  **Total cholesterol (TC, mg/dL)**  **T4 TC**  **T6 TC**  **High-density lipoprotein cholesterol (HDL, mg/dL)**  **T4 HDLC**  **T6 HDLC**  **Blood glucose (Glu, mg/dL)**  **T4 Glu**  **T6 Glu**  **Triglycerides (TG, mg/dL)**  **T4 TG**  **T6 TG** | 30.68 ± 3.67 (8)  27.32 ± 1.48 (8)    159.50 ± 14.25 (8)  69.00 ± 10.42 (8)    102.00 ± 13.01 (8)  42.00 ± 6.41 (8)    166.00 ± 20.73 (8) 200.00 ± 55.55 (8)    58.00 ± 12.47 (8)  45.50 ± 20.50 (8) | 46.98 ± 3.04 (9)  40.50 ± 2.97 (9)    200.89 ± 60.06 (9)  92.89 ± 15.59 (9)    128.00 ± 38.16 (9)  57.78 ± 7.77 (9)    207.56 ± 35.63 (9)  222.23 ± 34.88 (9)      65.78 ± 28.57 (9)  42.67 ± 17.20 (9) | 32.68 ± 4.42 (8)  28.15 ± 2.76 (8)    152.57 ± 16.24 (7)  75.00 ± 4.66 (8)    95.43 ± 12.31 (7)  45.50 ± 2.98 (8)    161.71 ± 26.42 (7)  217.00 ± 22.90 (8)      44.57 ± 9.36 (7)  32.50 ± 11.80 (8) | 45.46 ± 3.54 (9)  37.80 ± 3.07 (9)      203.11 ± 58.12 (9)  103.11 ± 10.73 (9)    121.33 ± 29.92 (9)  65.33 ± 2.83 (9)      219.11 ± 37.19 (9)  251.11 ± 39.38 (9)      46.22 ± 9.67 (9)  63.55 ± 18.16 (9) | 34.71 ± 5.67 (8)  29.21 ± 4.07 (8)    145.50 ± 19.94 (8)  87.00 ± 12.05 (8)      89.00 ± 12.05 (8)  46.00 ± 6.05 (8)    162.50 ± 22.42 (8)  257.50 ± 57.09 (8)      40.50 ± 8.12 (8)  79.50 ± 33.36 (8) | 44.10 ± 3.74 (9)  38.70 ± 4.95 (9)    213.56 ± 44.58 (9)  98.23 ± 21.74 (9)    125.11 ± 19.21 (9)  60.00 ± 7.75 (9)    204.00 ± 36.55 (9)  250.67 ± 63.28 (9)    54.67 ± 14.97 (9)  60.00 ± 24.00 (9) |

BCmw, bicarbonate- and calcium-rich mineral water; BCPMmw, bicarbonate-, calcium, potassium- and magnesium-rich mineral water; mwMix, alternation of natural mineral waters; n, number of animals; SD, standard deviation; T4, before any treatment; T6, after two months of treatment.

**Table 2S.** *Cont.*

| **Parameters** | **tap water-treated mice (control group)**  **tapw**  **Female (n) Male (n)** | |
| --- | --- | --- |
|  | **mean ± SD** | |
| **Body weight (BW, g)**  **T4 BW**  **T6 BW**  **Total cholesterol (TC, mg/dL)**  **T4 TC**  **T6 TC**  **High-density lipoprotein cholesterol (HDL, mg/dL)**  **T4 HDLC**  **T6 HDLC**  **Blood glucose (Glu, mg/dL)**  **T4 Glu**  **T6 Glu**  **Triglycerides (TG, mg/dL)**  **T4 TG**  **T6 TG** | 35.68 ± 4.19 (7)  30.36 ± 2.65 (7)  152.57 ± 10.18 (7)  85.43 ± 20.68 (7)      89.14 ± 8.55 (7)  49.57 ± 12.16 (7)    163.43 ± 29.88 (7)  234.43 ± 73.82 (7)      46.86 ± 6.82 (7)  47.29 ± 15.17 (7) | 46.65 ± 3.77 (9)  39.73 ± 3.67 (9)  206.67 ± 55.61 (9) 108.00 ± 26.46 (9)    123.11 ± 34.22 (9)  72.00 ± 12.49 (9)    240.00 ± 60.07 (9)  258.67 ± 69.74 (9)      47.55 ± 7.33 (9)  55.11 ± 29.51 (9) |

Tapw, low mineral content tap water; n, number of animals; SD, standard deviation; T4, before any treatment; T6, after two months of treatment.
